# Complete Genome Sequence of *Klebsiella pneumoniae* Strain ATCC 43816 KPPR1, a Rifampin-Resistant Mutant Commonly Used in Animal, Genetic, and Molecular Biology Studies

**DOI:** 10.1128/genomeA.00924-14

**Published:** 2014-09-25

**Authors:** Christopher A. Broberg, Weisheng Wu, James D. Cavalcoli, Virginia L. Miller, Michael A. Bachman

**Affiliations:** a Department of Microbiology & Immunology, University of North Carolina, Chapel Hill, North Carolina, USA; b BRCF Bioinformatics Core, University of Michigan, Ann Arbor, Michigan, USA; c Department of Computational Medicine and Bioinformatics, University of Michigan, Ann Arbor, Michigan, USA; d Department of Genetics, University of North Carolina, Chapel Hill, North Carolina, USA; e Department of Pathology, University of Michigan, Ann Arbor, Michigan, USA

## Abstract

*Klebsiella pneumoniae* is an urgent public health threat due to the spread of carbapenem-resistant strains causing serious, and frequently fatal, infections. To facilitate genetic, molecular, and immunological studies of this pathogen, we report the complete chromosomal sequence of a genetically tractable, prototypical strain used in animal models.

## GENOME ANNOUNCEMENT

*Klebsiella pneumoniae* is a Gram-negative rod of the *Enterobacteriaceae* family and a common cause of urinary tract infections, pneumonia, and bloodstream infections in hospitalized patients (1). Recently, a new clinical syndrome of pyogenic liver abscess and endophthalmitis emerged (2). The Centers for Disease Control and Prevention and the World Health Organization recently identified carbapenem-resistant *Enterobacteriaceae* (CRE), and specifically *K. pneumoniae*, as a significant public health threat not only due to extremely drug resistant (XDR) and pan-drug resistant (PDR) strains but also to the ease with which *K. pneumoniae* is able to transfer this drug resistance to other Gram-negative bacteria through horizontal gene transfer (3–5).

*K. pneumoniae* has been used extensively as a model organism to study the host response to Gram-negative pneumonia, including seminal research in Th17 biology and chemokine function (6, 7). Many of these studies have used strain ATCC 43816 as it recapitulates acute pneumonia with fatal systemic spread at a relatively low infectious dose. To facilitate genetic approaches to identify virulence genes in ATCC 43816, a rifampin-resistant-derivative (KPPR1 or VK055) was isolated. This genetically tractable strain has enabled random and site-directed mutagenesis to carefully study virulence factors including capsule and siderophores (8–11). However, additional studies are needed to elucidate not only the mechanisms *Klebsiella* uses to survive and flourish in the host, but also to provide a better understanding of the host immune response so that new therapies can be developed to circumvent the acute pathogenicity, increasing antibiotic resistance, and worldwide spread of new sequence types of this pathogen.

To facilitate further study into *Klebsiella* pathogenesis and the host response, the complete genome sequence of strain ATCC 43816 KPPR1 was determined. Sequencing was performed at the University of Michigan DNA Sequencing Core facility using a Pacific Biosciences PacBio RSII sequencer. A total of 901,752 reads, averaging 1.7 kb in length, were obtained for a total of 1.54 gigabases of sequence. Further sequencing was performed at the University of North Carolina (UNC) High Throughput Sequencing Facility (HTSF) using an Illumina HiSeq 2000 instrument generating 2 × 100 bp paired-end reads. A total of 53,036,370 reads were obtained for a total of 5.30 gigabases.

The University of Michigan Bioinformatics Core generated a *de novo* sequence assembly using the HGAP (Hierarchical Genome Assembly Process) protocol on SMRT Portal v2.2.0 to create an assembly of PacBio reads. A second independent whole-genome shotgun (WGS) assembly was generated at UNC using CLC Genomic Workbench v5.5.1 with the Illumina reads. The fidelity of the complete PacBio generated assembly was validated by performing a whole-genome alignment of the two assemblies using Mauve (12). The assembly resulted in a single circular chromosome with a G+C content of 57.4%, for a total of 5,374,834 bp. No plasmids were identified. The chromosome was annotated using the CloVR standard (r2014-05-12) pipeline (13) on the DIAG server followed by manual curation, resulting in 5,191 predicted genes, including 25 rRNA, 85 tRNA, and 5,081 protein coding sequences.

### Nucleotide sequence accession number.

This complete genome sequence has been deposited at GenBank under the accession no. CP009208.
